# Prolonged Diuretic, Natriuretic, and Potassium- and Calcium-Sparing Effect of Hesperidin in Hypertensive Rats

**DOI:** 10.3390/plants14091324

**Published:** 2025-04-27

**Authors:** Sabrina Lucietti Dick Orengo, Rita de Cássia Vilhena da Silva, Anelise Felício Macarini, Valdir Cechinel Filho, Priscila de Souza

**Affiliations:** Programa de Pós-Graduação em Ciências Farmacêuticas, Universidade do Vale do Itajaí, Itajaí 88302-901, SC, Brazil

**Keywords:** polyphenol, diuresis, hypertension, renoprotection

## Abstract

Systemic hypertension is a major global health concern, significantly contributing to the risk of cardiovascular, cerebrovascular, and renal diseases. Antihypertensive medications play a crucial role in lowering blood pressure, with diuretics serving as a particularly effective first-line therapy. However, the development of new compounds with diuretic properties, renal protective effects, and unique mechanisms of action remains a critical area of research for improving clinical outcomes. In this context, the present study investigated the diuretic and renal protective potential of the citrus flavonoid hesperidin in rats. Male spontaneously hypertensive and normotensive rats were treated with hesperidin at a dose of 3.0 mg/kg daily for seven days. Urine samples were analyzed for electrolytes (Na^+^, K^+^, Cl^−^, and Ca^2+^), biochemical parameters, and crystal precipitation, while renal tissues were examined histologically. Hesperidin treatment resulted in significant diuretic and natriuretic effects, along with potassium- and calcium-sparing properties. Furthermore, a marked reduction in calcium oxalate crystal formation was observed in the hesperidin-treated group. Histological analysis indicated a protective effect on renal tissue, with structural preservation observed in hypertensive rats. Docking studies revealed that hesperetin, the active metabolite of hesperidin formed upon oral administration, exhibited a high binding affinity for the calcium-sensing receptor (CaSR). This hypothesis may explain its role in preventing urinary crystalluria and contributing to calcium-sparing effects.

## 1. Introduction

Systemic hypertension (SH) ranks among the leading causes of morbidity and mortality in many countries worldwide. It is multifactorial in origin, and often asymptomatic treatment and prevention include adopting non-pharmacological strategies based on lifestyle modification, often combined with pharmacological treatment [1,2]. When necessary, pharmacological therapy should be initiated in cases of an uncontrolled systolic blood pressure of 140 mmHg or higher and/or diastolic blood pressure of 90 mmHg or higher [3,4]. Left uncontrolled over the years, SH can lead to various cardiovascular and renal alterations, potentially resulting in severe events such as heart failure, angina, myocardial infarction, arrhythmias, and renal insufficiency and failure [1,2,3,4].

Despite the wide availability of antihypertensive agents, hypertensive cardiovascular problems still affect millions today worldwide [1,2,3,4]. In the present context, diuretics have been used for more than 40 years in the treatment of SH, acting by raising the production of urine, which enhances the elimination of the excessive fluid and salts by the body. They remain one of the five first-line antihypertensive drug classes, with their efficacy proven in reducing morbidity and mortality related to the disease, especially thiazides and related agents [5].

Additionally, diuretics have shown their efficacy in many other disorders, such as liver cirrhosis, acute or chronic renal failure, nephrotic syndrome, and congestive heart failure, which, therefore, decreases the risk of cardiovascular events. However, given the large number of adverse reactions associated with disturbances in electrolytes and metabolism hypokalemia, calcium balance, glucose metabolism, hypercholesterolemia, hyperuricemia, ototoxicity, impotence, and gynecomastia, among others [5,6]. The development of more effective and safer diuretics is still desirable and important for clinical use. Recent studies show that natural products play a significant role in the development of new drugs [7], many of which may have biological activity. This perspective has led to growing interest in foods and plants rich in polyphenolic compounds, particularly flavonoids. Citrus flavonoids possess antioxidant effects through direct elimination of free radicals, as well as anti-inflammatory and hypoglycemic properties [8,9]. Among them, hesperidin, a glycoside bioflavonoid found in citrus fruits, stands out, with well-established biological properties, including antioxidant and anti-inflammatory effects [10,11], anticancer effects [12], antihyperglycemic effects [9], and cardiovascular protective properties [13,14].

Despite the range of described pharmacological properties and the extensive practical application of hesperidin, there is currently no preclinical or clinical data supporting a potential long-term diuretic effect of this flavanone. Our previous study has described the acute diuretic action of hesperidin in hypertensive rats [15], opening up possibilities for continued research along these lines. Considering all the demonstrated benefits of this compound, the present study aimed to evaluate the prolonged diuretic action of hesperidin in spontaneously hypertensive rats, with the expectation of contributing to the understanding of hesperidin’s renal effects and opening new possibilities for the management of SH and renal diseases.

## 2. Results

### 2.1. Prolonged Diuretic Effect of Hesperidin in Hypertensive Male Rats

As an initial strategy to validate the hypertension model and establish baseline physiological differences, we compared the systolic and diastolic blood pressure values between normotensive (NTRs) and spontaneously hypertensive rats (SHRs). Blood pressure measurements confirmed a significant difference between the groups, with SHRs displaying higher systolic and diastolic values compared to NTRs. Specifically, the systolic blood pressure was 170.0 ± 4.97 mmHg in SHRs and 119.4 ± 1.98 mmHg in NTR, while the diastolic blood pressure was 135.9 ± 3.29 mmHg in SHRs and 101.6 ± 1.49 mmHg in NTR.

**Figure 1:**
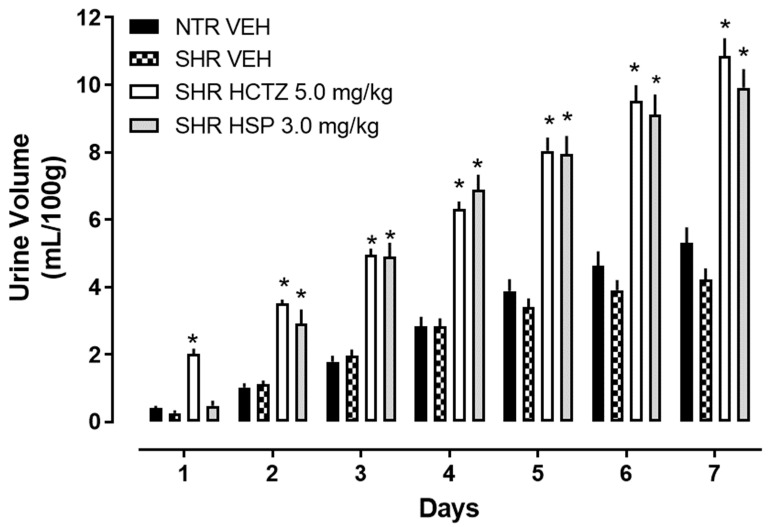
Urinary volume of spontaneously hypertensive (SHRs) rats treated with hesperidin (HSP). The values are expressed as mean ± SEM. Statistical analysis was performed using a two-way ANOVA followed by Dunnett’s multiple comparisons test. * *p* < 0.05 compared to the SHR VEH (vehicle). HCTZ = hydrochlorothiazide.

The analysis of the data obtained from the prolonged diuresis revealed that hesperidin, at a dose of 3.0 mg/kg orally given for 7 days, once per day, significantly increased urinary volume compared to the vehicle group. This result was comparable to the positive control group receiving HCTZ, as shown in Figure 1. This finding further emphasizes the diuretic characteristic of hesperidin and indicates that it exerts this effect over a prolonged period.

### 2.2. Prolonged Natriuretic and Potassium- and Calcium-Sparing Effects of Hesperidin

**Figure 2:**
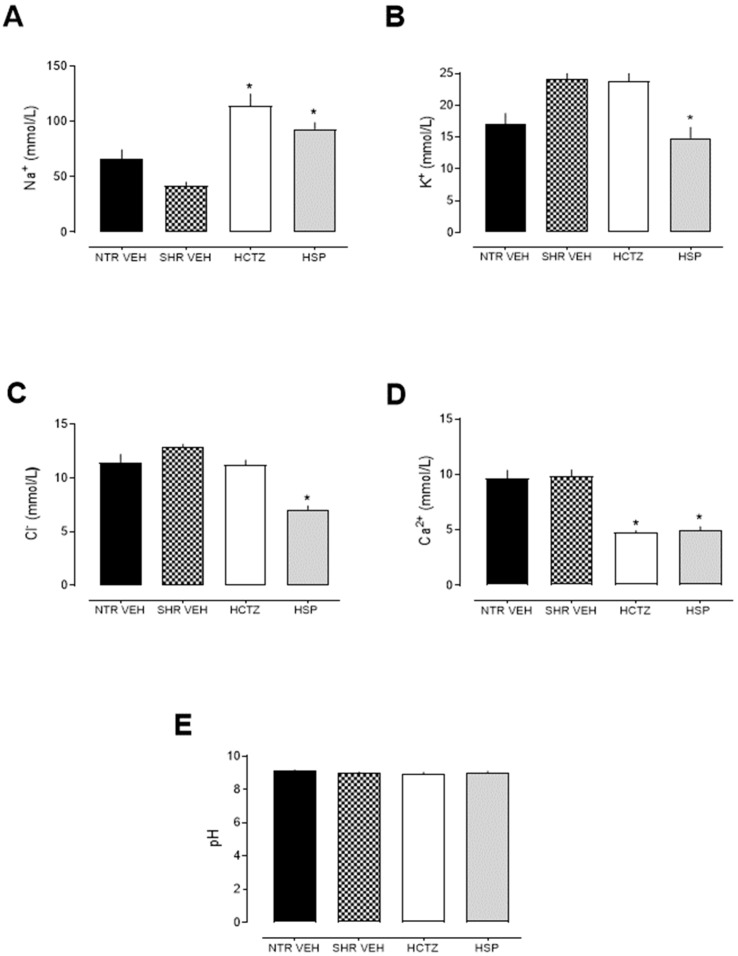
Analysis of urinary electrolytes Na^+^, K^+^, Cl^−^, Ca^2+^, and pH after oral treatment with hesperidin (HSP). (**A**) Analysis of urinary Na^+^. (**B**) Analysis of urinary K^+^. (**C**) Analysis of urinary Cl^−^. (**D**) Analysis of urinary Ca^2+^. (**E**) Analysis of urinary pH. Statistical analysis was performed using one-way ANOVA followed by Dunnett’s multiple comparisons test. * *p* < 0.05 when compared with SHR VEH (vehicle). HCTZ = Hydrochlorothiazide.

The animals treated with hesperidin showed a natriuretic effect (Figure 2A), highlighting the importance of sodium elimination for the diuretic effect. Moreover, the hesperidin-treated group exhibited reduced potassium excretion levels compared to the vehicle group (Figure 2B), suggesting that the long-term use of hesperidin might be a potential potassium-sparing agent, with a mechanism of action different from that of classic diuretics (both thiazide and loop diuretics). Hesperidin also decreased urinary Cl^−^ and Ca^2+^ levels (Figure 2C,D). Hesperidin demonstrated a natriuretic effect (Figure 2A), highlighting the importance of sodium elimination for the diuretic effect.

The reduction in chloride excretion after the use of diuretics may occur due to compensatory mechanisms of the kidney or due to direct action of the diuretic in certain tubular segments, such as the collecting tubules or in response to changes in acid–base balance. However, regarding pH values, no differences were observed between the groups (Figure 2E), suggesting that mechanisms related to acid–base balance do not appear to be involved in the actions of hesperidin. Instead, altered electrochemical gradients or regulatory adjustments in chloride transport may explain the observed effect, highlighting the need for further investigation into the precise tubular sites and pathways affected by hesperidin.

### 2.3. Analysis of Urinary Biochemical Parameters

**Figure 3:**
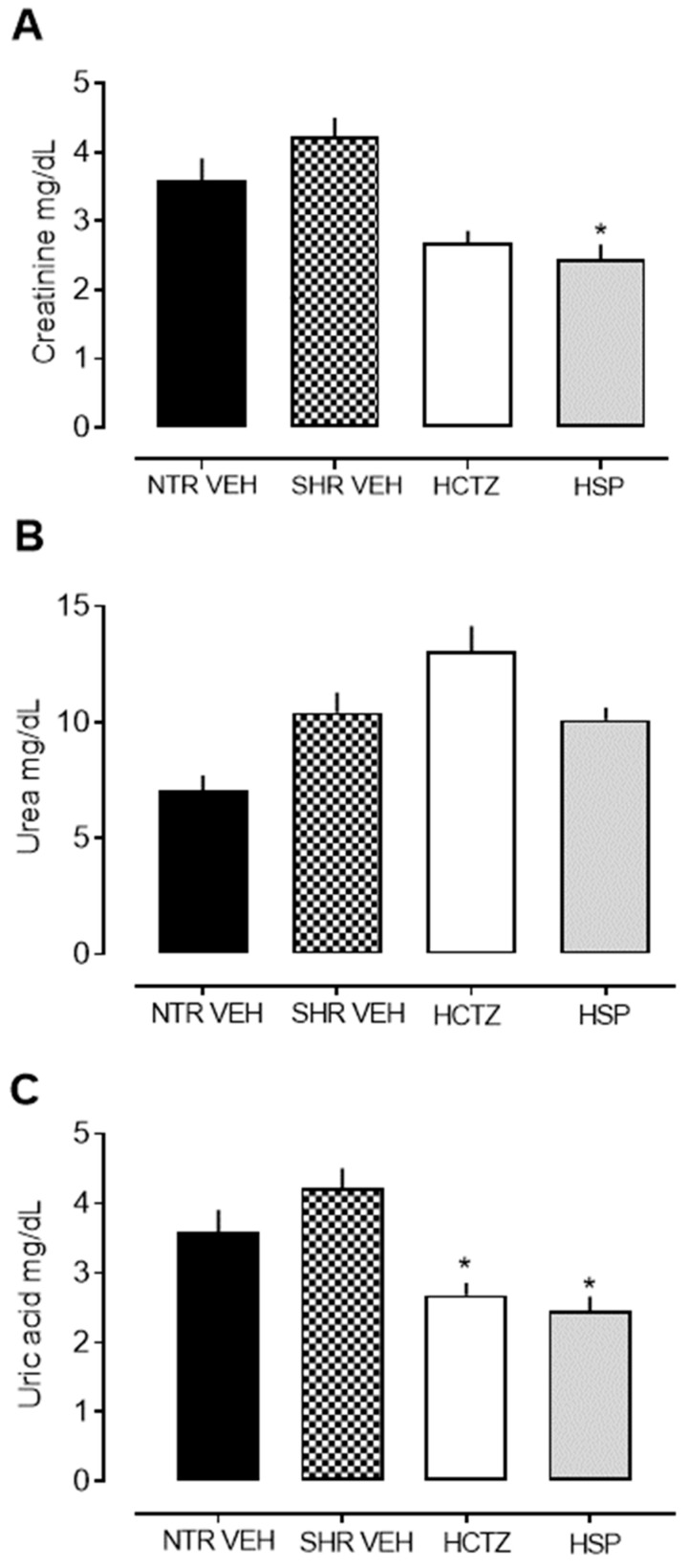
Analysis of urinary levels of urea, creatinine, and uric acid. (**A**) Creatinine levels. (**B**) Urea levels. (**C**) Uric acid levels. Statistical analysis was performed using one-way ANOVA followed by Dunnett’s multiple comparisons test. * *p* < 0.05 when compared with SHR VEH (vehicle). HCTZ = Hydrochlorothiazide.

As illustrated in Figure 3A–C, it was observed that hesperidin administered in repeated doses reduced creatinine and uric acid levels in the urine. Regarding urea elimination, no statistical difference was observed between the groups.

### 2.4. Analysis of Plasmatic Parameters

Table 1 presents the plasmatic parameters of the experimental groups. The parameters analyzed did not show statistically significant differences between the experimental groups, indicating that neither hypertension nor the treatments (HCTZ and HSP) substantially altered electrolyte balance or renal function markers. The values of Na^+^, K^+^, Cl^−^, and Ca^2+^ remained within a similar range across all groups, suggesting that their homeostasis was maintained despite the experimental conditions. The levels of creatinine, urea, and uric acid did not differ significantly among groups, indicating preserved renal function.

### 2.5. Assessment of Crystal Formation in Urine

**Figure 4:**
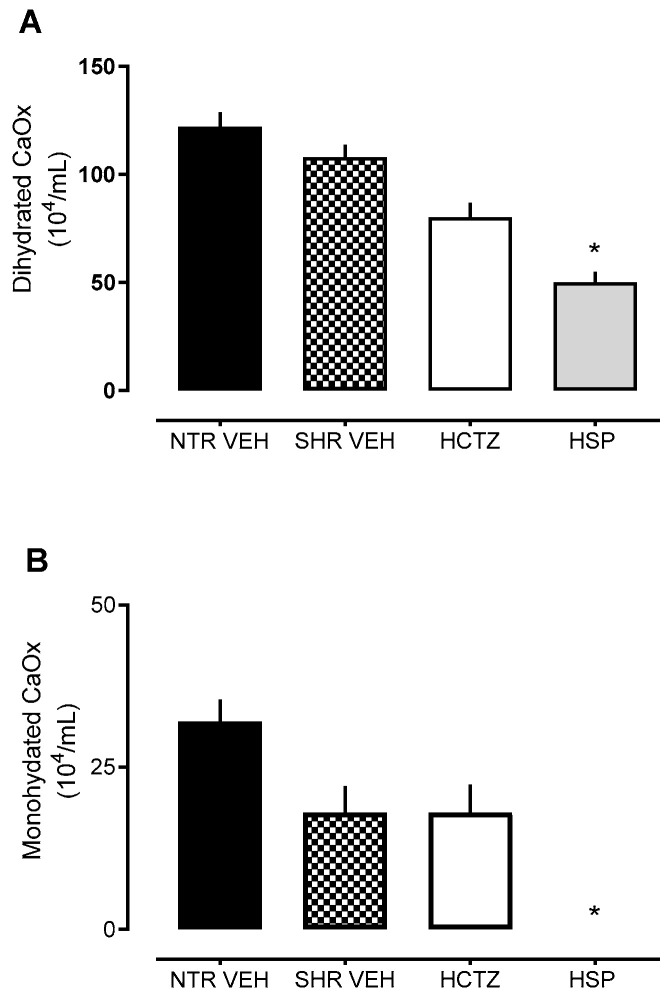
Analysis of CaOx crystal formation after prolonged diuresis. (**A**) Analysis of the dihydrated CaOx crystals in urine. (**B**) Analysis of the monohydrated CaOx crystals in urine. Statistical analysis was performed using one-way ANOVA followed by Dunnett’s multiple comparisons test. * *p* < 0.05 when compared with SHR VEH (vehicle). HCTZ = Hydrochlorothiazide.

**Figure 5:**
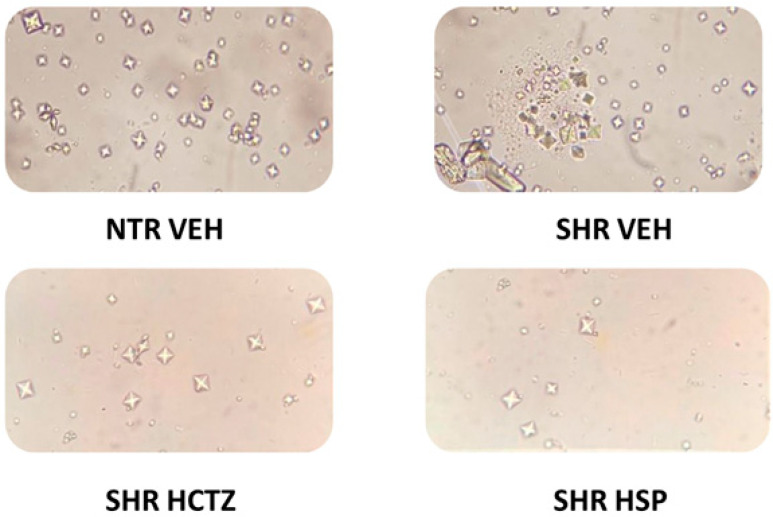
Representative images of the CaOx crystal formation in the urine after prolonged diuresis. NTR VEH: Normotensive rats treated with vehicle; SHR VEH: Hypertensive rats treated with vehicle; SHR HCTZ: Hypertensive rats treated with hydrochlorothiazide 5 mg/kg; SHR HSP: Hypertensive rats treated with hesperidin 3 mg/kg.

Values were analyzed regarding the effect of hesperidin in inhibiting the formation of dihydrated and monohydrated calcium oxalate crystals, as demonstrated in Figure 4 and Figure 5. There was a reduction in crystal formation in the urine of SHRs treated with hesperidin compared to the vehicle group. These data support the previously described results in this study, where hesperidin showed a calcium-sparing effect, contributing to the inhibition of crystal precipitation.

### 2.6. Histological Evaluation

**Figure 6:**
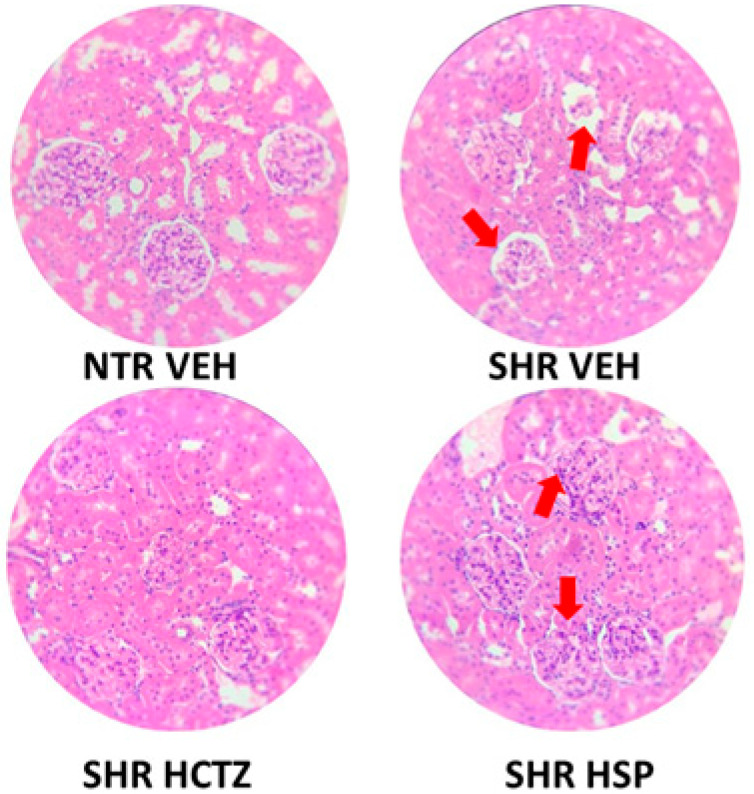
Representative histological images of the kidney sections obtained from animals after prolonged diuresis. NTR VEH: Normotensive rats treated with vehicle; SHR VEH: Hypertensive rats treated with vehicle; SHR HCTZ: Hypertensive rats treated with hydrochlorothiazide 5 mg/kg; SHR HSP: Hypertensive rats treated with hesperidin 3 mg/kg. The red arrows indicate greater corpuscle damage in the SHR VEH group and improved structure in the HSP-treated group.

As a qualitative analysis, a histological visualization of the kidney was performed to identify possible structural alterations. In Figure 6, it is observed that the SHR group exhibits greater distortion in the corpuscle indicated by the red arrow compared to the NTR group. It was also noted that the SHR group treated with hesperidin showed greater protection of the corpuscle (better organization of the mesangial space and the Bowman’s capsule) compared to the SHR-treated vehicle group, indicated by the red arrow, suggesting that hesperidin may act as a potential renal protector.

**Figure 7:**
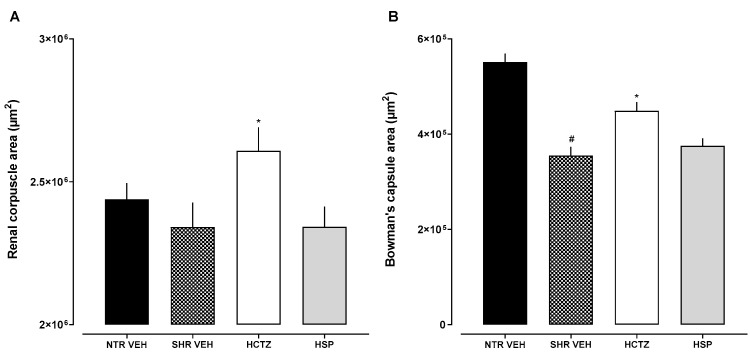
Histological analysis of the kidney sections obtained from animals after prolonged diuresis. (**A**) Renal corpuscle area; (**B**) Bowman’s capsule area. NTR VEH: Normotensive rats treated with vehicle; SHR VEH: Hypertensive rats treated with vehicle; SHR HCTZ: Hypertensive rats treated with hydrochlorothiazide 5 mg/kg; SHR HSP: Hypertensive rats treated with hesperidin 3 mg/kg. * *p* < 0.05 when compared with SHR VEH; # *p* < 0.05 when compared with NTR VEH.

Additionally, as a morphometric analysis, the sections demonstrated distinct changes among the experimental groups. A significant increase in both the renal corpuscle area and Bowman’s capsule area was observed only in hypertensive rats treated with hydrochlorothiazide (SHR HCTZ) when compared to the other groups. No significant changes were found in the group treated with hesperidin (SHR HSP). Additionally, when comparing normotensive rats (NTR VEH) to hypertensive untreated rats (SHR VEH), a significant reduction in Bowman’s capsule area was found in the SHR VEH group, with no difference in the renal corpuscle area (Figure 7).

### 2.7. Molecular Docking

The molecular docking results are presented in Table 2. The best predicted binding affinity was obtained for the calcium-sensing receptor in the Venus flytrap (VFT) domain for hesperetin. Although hesperidin exhibited better binding affinity than hesperetin in almost all the enzymes tested, only the hesperetin results were considered, as hesperidin is absorbed in its aglycone form (hesperetin) when taken orally.

Glycolate oxidase (GO) catalyzes the FMN-dependent oxidation of glycolate to glyoxylate, which is subsequently converted to oxalate, a key metabolite implicated in kidney stone formation. During docking studies of potential inhibitors, flavin mononucleotide (FMN) is maintained in the enzyme, as it serves as an essential prosthetic group in GO, crucial for the transformation of glycolate. Glycolate interacts with GO through two hydrogen bonds, involving Tyr26 and Arg263, as well as two additional hydrogen bonds with FMN. For inhibition, the most essential HB interactions involve Tyr26, Arg167, and Arg263, and hydrophobic interactions involve Leu205, Val209, and Trp110 [16]. In the docking analysis, Hesperetin was predicted to form HB with Tyr132 and Arg167, along with hydrophobic interactions involving Trp110, Met82, Leu164, Leu205, Ala81, His260, and the FMN prosthetic group.

Phosphoethanolamine cytidylyltransferase (PEC) is an enzyme that regulates the biosynthesis of phosphatidylethanolamine (PtdEtn) that was identified as a possible promoter of the formation of calcium oxalate crystals by enhancing its nucleation and growth [17]. PtdEtn synthesis from ethanolamine by PEC generates glyoxal, a precursor involved in oxalic acid production, suggesting a link between this enzyme and stone formation. A molecular modeling study revealed that key amino acids, including His226, His229, His307, Thr310, and Thr342, are important for ligand binding within the enzyme’s binding pocket. Notably, Val218, Ala219, Tyr258, and Leu 340 were involved in hydrophobic interactions and Ala221 and Ser336 played a role in hydrogen bonding [18].

Hepesperetin presented predicted HB with His226, His229, Leu340, and Ser336 alongside polar interactions with Lys259, His307, Thr310, etc. and hydrophobic interactions with Ala219, Tyr258, Thr342, etc.

The CaSR is a family C G protein-coupled receptor (GPCR) that is highly expressed in the parathyroid glands and kidneys [19]. It maintains systemic Ca^2+^ balance by sensing elevated blood Ca^2+^ levels, triggering intracellular signaling that reduces parathyroid hormone (PTH) secretion and decreases renal calcium reabsorption [19,20,21]. This process, however, can increase urinary calcium levels, potentially exacerbating kidney stone formation. Its endogenous ligands, including Ca^2+^ and Mg^2+^ ions as well as L-amino acids like L-tryptophan, bind to the extracellular (EC) domain, typically within a ligand-binding region known as the Venus flytrap (VFT) domain, which is connected to the 7-transmembrane (7TM) domain by a cysteine-rich domain [21].

**Figure 8:**
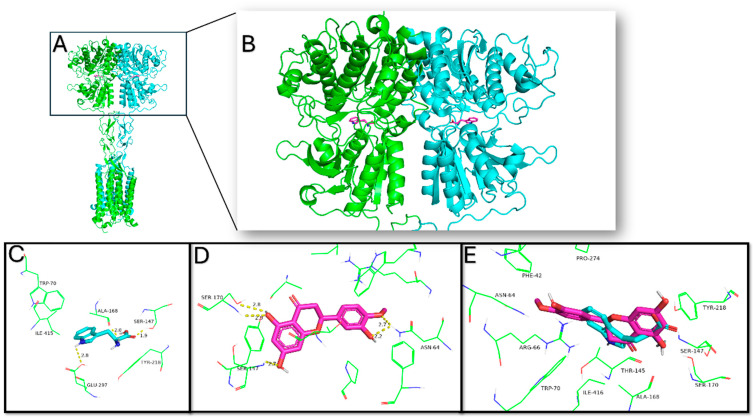
The CaSR (**A**), highlighting the extracellular domain with the L-tryptophan residue in the VFT domain (**B**); L-tryptophan and its interacting amino acids (**C**); hesperetin and its predicted amino acid interactions (**D**); and the overlap of L-tryptophan and hesperetin in the binding site (**E**).

In the docking analysis with the L-amino acid binding site, in the VTF domain, hesperetin presented the best binding affinity, and its pose in the binding site is presented in Figure 8. It was predicted that hydrophobic interactions occur with Trp70, Thr145, Ala168, Tyr218, and Pro274 and also hydrogen bonds with Ser147, Ser170, and Asn64. The endogenous ligand, L-tryptophan, presents hydrogen bond interactions with Ser147, Ser170, Ala168, and Glu297, and hydrophobic interactions are formed with Trp70 and Ile416. Also, Etelcalcetide, a distinctive CaSR positive allosteric modulator (PAM), is predicted to bind to the CaSR VFT domain by forming a disulfide bond with Cys482, which is situated close to the “hinge” region in the VFT lobe 1 (LB1), a critical area involved in VFT closure [22].

When docked to the 7TM domain, hesperetin was predicted to form hydrophobic interactions with Phe683, Phe687, Phe813, Trp817, and Ile776, along with hydrogen bonds involving Gln680 and Gly684. Clinically, the PAM molecule evocalcet, used in the treatment of secondary hyperparathyroidism, is bound to the 7TM domain of the CaSR by hydrophobic interactions with residues Phe683, Phe687, Trp817, and Ile840. The nitrogen atom in the center position makes two hydrogen bonds with residues Glu836 and Gln680 [20]. The negative allosteric modulator (NAM) molecule NPS-2143 is positioned within a hydrophobic pocket formed by residues Ile840, Phe683, Ile776, Trp817, Leu772, Tyr824, and Phe820 and is stabilized through three hydrogen bonds with Glu836 [20]. However, docking analysis alone cannot determine whether it acts as a positive or negative modulator; thus, additional studies are required to confirm its modulatory effects.

## 3. Discussion

Biological activities derived from plant species have shown significant benefits in the treatment of pathologies affecting the circulatory system and kidneys. Hypertension is challenging to manage, and there is a shortage of drugs with minimal adverse effects and enhanced biological activity. In fact, in most cardiac and renal pathologies, diuretics are generally present. Their administration is typically combined with other drugs and aims to increase the concentration of Na^+^ and water in the renal tubules to induce diuresis. They are commonly used to treat conditions such as hypertension, congestive heart failure, edema, and kidney disease. Diuretics are classified into five categories based on their efficacy, mechanism of action, and location in the nephron [23]. Various plant-derived compounds have been reported as diuretic in experimental studies [24,25]. However, despite the numerous pharmacological activities already described for hesperidin, this is the first report of its potential diuretic effect after a dose-repeated treatment.

The findings of the present study demonstrated that hesperidin, administered daily at a dose of 3 mg/kg, significantly increased urinary volume and Na^+^ excretion compared to the vehicle. The diuretic data were similar to the values obtained with hydrochlorothiazide, a thiazide diuretic used as a control. Recent studies show that natriuretic function, as observed with hesperidin administration in this study, is the mechanism responsible for long-term blood pressure control, adjusting and setting the basal level of blood pressure. Thus, Na^+^ excretion or reabsorption is responsible for transient variations in blood pressure [26]. Therefore, this mechanism adjusts blood volume to regulate blood pressure, making electrolyte balance in sodium levels crucial for hypertension management. It is worth noting that this study was conducted entirely in hypertensive animals, which are the most commonly used models for hypertension [27].

It was also observed that the animals treated with hesperedin showed reduced potassium excretion levels compared to the vehicle, suggesting that the long-term use of hesperidin may act as a potential potassium-sparing agent, which is a significant point of distinction from HCTZ. These data were not observed in the acute study [15], reinforcing the importance of studies with repeated doses to confirm the effects. These data are also crucial because many adverse effects of diuretics are related to excessive K^+^ loss in urine.

Based on these data, it is suggested that hesperidin acts through a mechanism different from classical diuretics (both thiazide and loop diuretics). Thiazide and loop diuretics have higher diuretic power but cause excessive K^+^ loss, while potassium-sparing diuretics, despite the benefit of preserving this electrolyte, have low diuretic power. Thus, hesperedin appears as a promising alternative, as it preserves K^+^ and has a diuretic power that is comparable to the standards used. It is important to note that the concentrations of Na^+^, K^+^, Cl^−^, and Ca^2+^ in the blood remained consistent across all experimental groups, indicating that electrolyte homeostasis was effectively maintained regardless of the treatment or condition applied. Similarly, there were no significant differences in creatinine, urea, and uric acid levels among the groups, suggesting that, despite the changes seen in the urine, renal function was preserved.

Calcium oxalate crystals are microscopic, insoluble crystals composed of calcium and oxalate, and they can exist in different forms, including monohydrated and dihydrated. These forms refer to the number of water molecules associated with the crystalline structure [28]. These crystals can form in the urine, and, when present, they are one of the main components of kidney stones. To elucidate the factors influencing crystal formation and explore mechanisms to modulate these processes, discovering new approaches for managing kidney stone disease becomes relevant. Urolithiasis involves a physiological change in the natural crystallization conditions of urine. When crystals accumulate and grow, they can form stones that block urinary flow, causing intense pain and even urinary infections [29]. There are no clinical reports addressing strategies that are entirely effective for dissolving these crystals.

Based on this, values related to the effect of hesperidin in inhibiting the formation of dihydrated and monohydrated calcium oxalate crystals were analyzed in urine collected from animals treated daily with hesperidin. Hesperidin was able to decrease crystal formation, a piece of data that corroborates the results previously described in this study, where hesperidin demonstrated a calcium-sparing effect, as reducing calcium excretion can be an important strategy in patients with a higher likelihood of forming kidney stones due to calcium crystallization in urine [30].

Docking analysis was performed since, while factors such as urinary pH, calcium excretion, fluid intake, and diet are well-established contributors to kidney stone formation, growing research highlights the role of specific protein receptors and enzymes directly or indirectly involved in the pathophysiology of urolithiasis. These proteins may significantly influence key pathways in crystal nucleation, growth, and aggregation [31].

For instance, enzymes involved in oxalate production, such as glycolate oxidase (GO) [16] and phosphoethanolamine cytidylyltransferase (PEC) [32], as well as receptors involved in calcium homeostasis like the calcium-sensing receptor (CaSR) [31], play critical roles in urolithiasis. In the docking analysis targeting the L-amino acid binding site within the VFT domain, hesperetin demonstrated the highest binding affinity, interacting at a site similar to that of L-tryptophan. These interactions suggest a potential role in maintaining the receptor’s active state, which may enhance renal calcium reabsorption—a possible mode for kidney stone management—and, also, corroborate the calcium-sparing effects previously observed [33,34].

The docking results presented in this study provide valuable hypotheses and suggest a potential mechanism of action for hesperidin—and its bioactive metabolite, hesperetin (formed following oral administration)—in the prevention of kidney stone formation. These findings offer a foundation for further experimental and mechanistic investigations into the therapeutic potential of these compounds in nephrolithiasis management.

Lastly, the present study performed a histological assessment of the kidney to identify potential structural changes. Similar to humans, hemodynamic and metabolic disorders in SHRs are also revealed through multifactorial pathways. Hypertensive injury in SHRs is related to sustained elevated blood pressure, with vascular impairment causing arterial hypertrophy in the juxtamedullary cortex of the renal tissue. The progression of arteriolar hypertrophy, in turn, leads to the failure of some glomerular mesangial regions and tubular atrophy, reducing glomerular filtration [35]. Our data show this differentiation, which the reduced Bowman’s capsule area observed in hypertensive untreated animals compared to normotensive controls suggests early glomerular impairment associated with uncontrolled hypertension. It was also observed that the SHR group treated with hesperidin resulted in improved protection of the corpuscle (greater organization of the mesangial space and Bowman’s capsule), suggesting that hesperidin may act as a potential renal protector. Studies demonstrate that hesperidin helps to mitigate renal damage caused by factors such as ischemia-reperfusion injury, nephrotoxic agents, and diabetic nephropathy by preserving renal function, reducing renal injury markers, and improving organ functionality [36], corroborating the data found in the present work. Moreover, the observed increase in renal corpuscle and Bowman’s capsule areas in the SHR HCTZ group suggests that the prolonged use of hydrochlorothiazide may induce morphological alterations in renal structures. These changes could be related to compensatory mechanisms resulting from chronic diuretic use or to direct adverse effects of the drug on kidney tissue. Taken together, these results reinforce the importance of exploring alternative treatments like hesperidin that may better preserve renal integrity.

## 4. Materials and Methods

### 4.1. Drugs and Reagents

Hesperidin and hydrochlorothiazide were obtained from Sigma-Aldrich Chemical Co. (St. Louis, MO, USA). Other substances were purchased from Merck (Darmstadt, Germany).

### 4.2. Animals

In this study, male normotensive and spontaneously hypertensive rats (SHR) aged between 12 and 16 weeks were used, provided by the University of Vale do Itajaí, after approval from the institutional ethics committee (authorization no. 020/22). The animals were housed in a standard environment with a 12 h light/dark cycle and a temperature of 22 ± 2 °C, with free access to food and water.

### 4.3. Assessment of Prolonged Diuresis

Before starting the experiments, systolic blood pressure (SBP) and diastolic blood pressure (DBP) were measured using plethysmography (Serial number: 007006; Bonther, Ribeirão Preto, SP, Brazil). To reduce the stress of the restraint required to measure blood pressure, the groups were trained to adapt the equipment in the week before the first blood pressure measurement. Following adaptation in a temperature-controlled room (28–30 °C), the animals were placed in acrylic restrainers and positioned on a heated platform. A pre-calibrated transducer was connected to a sphygmomanometer, which was placed around the animal’s tail and equipped with an automated inflation system. This system was integrated with a data acquisition and conversion unit, linked to a computer running specialized software (Tail Plethysmography.Ink, Bonther, Ribeirão Preto, SP, Brazil) for precise data recording and analysis.

For the assessment of prolonged diuresis, animals were randomly assigned to groups of 6 animals each: the vehicle (VEH; distilled water) group from NTRs and SHRs; and SHR groups treated orally with hydrochlorothiazide (HCTZ; 5 mg/kg) or hesperidin (HSP 3 mg/kg) once daily for 7 days. The animals were then housed in individual metabolic cages for 7 days. Urine was collected and expressed, cumulatively, per 100 g of body weight. The values of pH, electrolyte excretion (Na^+^, K^+^, Cl^−^, Ca^2+^), urea, creatinine, and uric acid were measured in each cumulative urine sample at the end of the experiment. The pH of the samples was assessed using a calibrated pH meter (Digimed, São Paulo, SP, Brazil). The levels of Na^+^ and K^+^ were determined using a flame photometer. Cl^−^, Ca^2+^, urea, creatinine, and uric acid concentrations were measured using commercial assay kits from Bioclin (Bioclin, Belo Horizonte, MG, Brazil), following the manufacturer’s instructions.

Furthermore, at the end of the 7-day experiment, the animals were anesthetized with a solution of xylazine (10 mg/kg) and ketamine (80 mg/kg), and renal tissue was collected for histological evaluation and plasma samples for biochemical parameters (following the same protocol described above). Histological analyses were performed on the left kidney, and images were captured to assess the renal corpuscle and Bowman’s capsule areas using ImageJ 1.38e software. Measurements were taken from randomly selected renal corpuscles, with approximately 10–15 measurements per group.

From the same urine collected at the end of the prolonged diuresis, the ex vivo urinary stone model was induced by the precipitation and formation of calcium oxalate crystals. The test followed these steps. Crystal precipitation was induced with 0.1 M sodium oxalate, administered at 40 µL per mL of urine, in all groups. After this step, urine was incubated for 60 min for crystal formation. After this period, the total number and differentiated (monohydrated and dehydrated forms) crystals in each group were evaluated in four randomly selected fields using a Neubauer chamber with 400× magnification under a microscope (Olympus CBA Microscope, Olympus Technology Services PTY LTD, Parramatta, NSW, Australia). All tests were performed in triplicate.

### 4.4. Statistical Analysis

The results are expressed as mean ± S.E.M. (n = 5–8 animals per group). Results were analyzed statistically by one-way or two-way ANOVA followed by the Dunnett’s test, accordingly. A *p*-value of less than 0.05 was considered significant for obtained results.

### 4.5. Molecular Docking

Molecular docking was performed for both hesperidin and hesperetin structures, since hesperidin when orally administered is absorbed as the aglycone form, hesperetin. Crystal structures of the enzymes chosen were retrieved from Protein Data Bank (PDB) [37]. The proteins were prepared using the AutoDock Tools (ADT 1.5.7) program [38]: co-crystal ligands, water, and ions were removed, hydrogens were added, and Gasteiger charges calculated. The 3D structures of hesperidin and hesperetin were retrieved in mol2 format from PubChem (National Center for Biotechnology Information, Bethesda, MD, USA) and prepared for docking using the ADT 1.5.7 program. Molecular docking was performed using AutoDock Vina [39]. The possible interactions and 3D images were generated using PyMOL 3.0.3 (Schrodinger LLC, 2015, New York, NY, USA) and 2D images were generated using the BIOVIA Discovery Studio program.

The enzymes were selected based on their potential interactions with urolithiasis pathogenesis. These include Glycolate Oxidase (PDB ID: 2RDT), the calcium-sensing receptor in the Venus flytrap (VFT) domain (PDB ID: 5FBK), the calcium-sensing receptor in the 7-transmembrane (7TM) domain (PDB ID: 7DD7), and Phosphoethanolamine Cytidylyltransferase (PDB ID: 3ELB).

All raw data supporting the findings described in this study are provided in the Supplementary Material.

## 5. Conclusions

Based on the findings, hesperidin, a flavonoid found in citrus fruits, demonstrated potential diuretic and renal protective effects in hypertensive rats after a dose-repeated treatment. The diuretic effect is justified by the increased urine volume and sodium elimination. It was also noted that hesperidin exhibited potassium- and calcium-sparing effects, an important feature that differentiates it from other clinically known diuretics. Additionally, hesperidin significantly reduced the formation of calcium oxalate crystals, which is attributed, at least in part, to the decreased calcium excretion in the urine. The docking results suggest potential molecular mechanisms underlying the activity of hesperidin, paving the way for further in-depth investigations into its mode of action.

## Figures and Tables

**Table 1:** Plasmatic biochemical parameters of experimental groups. Values are expressed as mean ± SEM. No statistically significant differences were observed between groups. VEH NTR: normotensive vehicle-treated group; VEH SHR: hypertensive vehicle-treated group; HCTZ: hydrochlorothiazide-treated group; HSP: hesperidin-treated group.

	VEH NTR	VEH SHR	HCTZ SHR	HSP SHR
Na^+^ (mmol/L)	137.50 ± 1.23	139.50 ± 0.54	137.00 ± 0.76	138.30 ± 0.15
K^+^ (mmol/L)	5.90 ± 0.34	5.58 ± 0.18	5.45 ± 0.04	5.51 ± 0.12
Cl^−^ (mmol/L)	97.14 ± 0.46	98.52 ± 0.18	98.66 ± 0.48	98.52 ± 0.41
Ca^2+^ (mmol/L)	1.42 ± 0.03	1.37 ± 0.01	1.36 ± 0.01	1.35 ± 0.01
Creatinine (mg/dL)	0.25 ± 0.03	0.23 ± 0.01	0.24 ± 0.01	0.22 ± 0.01
Urea (mg/dL)	59.64 ± 1.31	52.35 ± 0.91	54.35 ± 0.48	54.48 ± 0.99
Uric acid (mg/dL)	1.24 ± 0.09	1.03 ± 0.09	1.03 ± 0.09	1.16 ± 0.08

**Table 2:** Predicted molecular docking binding affinity of hesperetin and hesperidin with selected enzymes possibly involved with urolithiasis pathogenesis.

	Binding Affinity (kcal/mol)		
Enzyme	PDB ID	Hesperetin	Hesperidin
Glycolate Oxidase	2RDT	−7.5	−8.5
Calcium-sensing receptor VFT	5FBK	−9.2	−8.5
Calcium-sensing receptor 7TM	7DD7	−8.5	−9.6
Phosphoethanolamine Cytidylyltransferase	3ELB	−8.4	−8.7

## Data Availability

All raw data supporting the findings described in this study are provided in the Supplementary Material.

## Supplementary Materials

The following supporting information can be downloaded at: https://www.mdpi.com/article/10.3390/plants14091324/s1.

## References

[1] Mills K.T., Stefanescu A., He J. (2020). The global epidemiology of hypertension. Nat. Rev. Nephrol..

[2] Abu H., Aboumatar H., Carson K.A., Goldberg R., Cooper L.A. (2018). Hypertension knowledge, heart healthy lifestyle practices and medication adherence among adults with hypertension. Eur. J. Pers. Cent. Healthc..

[3] Thomopoulos C., Parati G., Zanchetti A. (2017). Effects of blood-pressure-lowering treatment on outcome incidence. 12. Effects in individuals with high-normal and normal blood pressure: Overview and meta-analyses of randomized trials. J. Hypertens..

[4] Tsioufis C., Thomopoulos C. (2017). Combination drug treatment in hypertension. Pharmacol. Res..

[5] Blowey D.L. (2016). Diuretics in the treatment of hypertension. Pediatr. Nephrol..

[6] Sica D.A. (2004). Diuretic-related side effects: Development and treatment. J. Clin. Hypertens..

[7] Newman D.J., Cragg G.M. (2020). Natural products as sources of new drugs over the nearly four decades from 01/1981 to 09/2019. J. Nat. Prod..

[8] Miler M., Živanović J., Ajdžanović V., Oreščanin-Dušić Z., Milenković D., Konić-Ristić A., Blagojević D., Milošević V., Šošić-Jurjević B. (2016). Citrus flavanones naringenin and hesperetin improve antioxidant status and membrane lipid compositions in the liver of old-aged Wistar rats. Exp. Gerontol..

[9] Constantin R.P., Constantin R.P., Bracht A., Yamamoto N.S., Ishii-Iwamoto E.L., Constantin J. (2014). Molecular mechanisms of citrus flavanones on hepatic gluconeogenesis. Fitoterapia.

[10] Pyrzynska K. (2022). Hesperidin: A Review on Extraction Methods, Stability and Biological Activities. Nutrients.

[11] Yang H.L., Chen S.C., Senthil Kumar K.J., Yu K.N., Lee Chao P.D., Tsai S.Y., Hou Y.C., Hseu Y.C. (2012). Antioxidant and anti-inflammatory potential of hesperetin metabolites obtained from hesperetin-administered rat serum: An ex vivo approach. J. Agric. Food Chem..

[12] Aggarwal V., Tuli H.S., Thakral F., Singhal P., Aggarwal D., Srivastava S., Pandey A., Sak K., Varol M., Khan M.A. (2020). Molecular mechanisms of action of hesperidin in cancer: Recent trends and advancements. Exp. Biol. Med..

[13] Roohbakhsh A., Parhiz H., Soltani F., Rezaee R., Iranshahi M. (2015). Molecular mechanisms behind the biological effects of hesperidin and hesperetin for the prevention of cancer and cardiovascular diseases. Life Sci..

[14] Maneesai P., Bunbupha S., Potue P., Berkban T., Kukongviriyapan U., Kukongviriyapan V., Prachaney P., Pakdeechote P. (2018). Hesperidin Prevents Nitric Oxide Deficiency-Induced Cardiovascular Remodeling in Rats via Suppressing TGF-β1 and MMPs Protein Expression. Nutrients.

[15] de Souza P., da Silva R.C.V., Mariano L.N.B., Dick S.L., Ventura G.C., Cechinel-Filho V. (2022). Diuretic and Natriuretic Effects of Hesperidin, a Flavanone Glycoside, in Female and Male Hypertensive Rats. Plants.

[16] Cabrera N., Cuesta S.A., Mora J.R., Paz J.L., Márquez E.A., Espinoza-Montero P.J., Marrero-Ponce Y., Pérez N., Contreras-Torres E. (2022). Searching glycolate oxidase inhibitors based on QSAR, molecular docking, and molecular dynamic simulation approaches. Sci. Rep..

[17] Aggarwal K.P., Tandon S., Naik P.K., Singh S.K., Tandon C. (2013). Peeping into Human Renal Calcium Oxalate Stone Matrix: Characterization of Novel Proteins Involved in the Intricate Mechanism of Urolithiasis. PLoS ONE.

[18] Chattaraj B., Nandi A., Das A., Sharma A., Dey Y.N., Kumar D. (2023). Inhibitory activity of Enhydra fluctuans Lour. on calcium oxalate crystallisation through in silico and in vitro studies. Front. Pharmacol..

[19] Hannan F.M., Kallay E., Chang W., Brandi M.L., Thakker R.V. (2019). The calcium-sensing receptor in physiology and in calcitropic and noncalcitropic diseases. Nat. Rev. Endocrinol..

[20] Wen T., Wang Z., Chen X., Ren Y., Lu X., Xing Y., Lu J., Chang S., Zhang X., Shen Y. (2021). Structural basis for activation and allosteric modulation of full-length calcium-sensing receptor. Sci Adv..

[21] Zhang C., Zhang T., Zou J., Miller C.L., Gorkhali R., Yang J.-Y., Schilmiller A., Wang S., Huang K., Brown E.M. (2016). Structural basis for regulation of human calcium-sensing receptor by magnesium ions and an unexpected tryptophan derivative co-agonist. Sci. Adv..

[22] Diao J., DeBono A., Josephs T.M., Bourke J.E., Capuano B., Gregory K.J., Leach K. (2021). Therapeutic opportunities of targeting allosteric binding sites on the calcium-sensing receptor. ACS Pharmacol. Transl. Sci..

[23] Kehrenberg M.C.A., Bachmann H.S. (2022). Diuretics: A contemporary pharmacological classification?. Naunyn-Schmiedeberg Arch. Pharmacol..

[24] de Souza P., Mariano L.N.B., Cechinel-Zanchett C.C., Cechinel-Filho V. (2021). Promising Medicinal Plants with Diuretic Potential Used in Brazil: State of the Art, Challenges, and Prospects. Planta Med..

[25] Mariano L.N.B., Boeing T., da Silva R.C.V., da Silva L.M., Gasparotto-Júnior A., Cechinel-Filho V., de Souza P. (2022). Exotic Medicinal Plants Used in Brazil with Diuretic Properties: A Review. Chem. Biodivers..

[26] Díaz-Morales N., Baranda-Alonso E.M., Martínez-Salgado C., López-Hernández F.J. (2023). Renal sympathetic activity: A key modulator of pressure natriuresis in hypertension. Br. J. Pharmacol..

[27] Pravenec M., Křen V., Landa V., Mlejnek P., Musilová A., Šilhavý J., Šimáková M., Zídek V. (2014). Recent progress in the genetics of spontaneously hypertensive rats. Physiol. Res..

[28] El Beze J., Mazeaud C., Daul C., Ochoa-Ruiz G., Daudon M., Eschwège P., Hubert J. (2022). Evaluation and understanding of automated urinary stone recognition methods. BJU Int..

[29] Ratkalkar V.N., Kleinman J.G. (2011). Mechanisms of Stone Formation. Clin. Rev. Bone Miner. Metab..

[30] Sorensen M.D. (2014). Calcium intake and urinary stone disease. Transl. Androl. Urol..

[31] Haque Z., Taleuzzaman M., Jamal R., Al-Qahtani N.H., Haque A. (2024). Targeting protein receptors and enzymes for precision management of urolithiasis: A comprehensive review. Eur. J. Pharmacol..

[32] Macarini A.F., Mariano L.N.B., Zanovello M., da Silva R.C.V., Corrêa R., de Souza P. (2024). Protective Role of Rosmarinic Acid in Experimental Urolithiasis: Understanding Its Impact on Renal Parameters. Pharmaceuticals.

[33] Shirfule A.L., Sangamwar A.T., Khobragade C.N. (2011). Exploring glycolate oxidase (GOX) as an antiurolithic drug target: Molecular modeling and in vitro inhibitor study. Int. J. Biol. Macromol..

[34] Aggarwal B.B., Yuan W., Li S., Gupta S.C. (2013). Curcumin-free turmeric exhibits anti-inflammatory and anticancer activities: Identification of novel components of turmeric. Mol. Nutr. Food Res..

[35] Hultström M. (2012). Development of structural kidney damage in spontaneously hypertensive rats. J. Hypertens..

[36] Kandhwal M., Grewal A.K., Singh M., Singh V., Singh T.G. (2024). Neuroprotective effects of hesperidin: In-Vitro and in silico evaluation of its antioxidant and enzyme inhibitory activities. Carpath. J. Food Sci. Technol..

[37] Berman H.M., Westbrook J., Feng Z., Gilliland G., Bhat T.N., Weissig H., Shindyalov I.N., Bourne P.E. (2000). The Protein Data Bank. Nucleic Acids Res..

[38] de Souza V.T., de Franco É.P.D., de Araújo M.E.M.B., Messias M.C.F., Priviero F.B.M., Frankland Sawaya A.C., de Oliveira Carvalho P. (2016). Characterization of the antioxidant activity of aglycone and glycosylated derivatives of hesperetin: An in vitro and in vivo study. J. Mol. Recognit..

[39] Trott O., Olson A.J. (2010). AutoDock Vina: Improving the speed and accuracy of docking with a new scoring function, efficient optimization, and multithreading. J. Comput. Chem..

