# Supplementary material for: Prolonged Diuretic, Natriuretic, and Potassium- and Calcium-Sparing Effect of Hesperidin in Hypertensive Rats
Source: Plants (Basel). 2025 Apr 27;14(9):1324. doi: 10.3390/plants14091324 (PMC12073609; doi:10.3390/plants14091324)
Supplement: Supplementary file 1 [file plants-14-01324-s001.zip › plants-3549272-dataset-file.pdf]

Table S1. Predicted molecular docking binding affinity of hesperetin and hesperidin with selected enzymes possibly involved with urolithiasis pathogenesis.

		Binding affinity (kcal/mol)	
**Enzyme**	**PDB ID**	**Hesperetin**	**Hesperidin**
**Glycolate Oxidase**	2RDT	-7.5	-8.5
**Calcium-sensing receptor VFT**	5FBK	-9.2	-8.5
**Calcium-sensing receptor 7TM**	7DD7	-8.5	-9.6
**Phosphoethanolamine Cytidylyltransferase**	3ELB	-8.4	-8.7
