# Supplementary material for: Prolonged Diuretic, Natriuretic, and Potassium- and Calcium-Sparing Effect of Hesperidin in Hypertensive Rats
Source: Plants (Basel). 2025 Apr 27;14(9):1324. doi: 10.3390/plants14091324 (PMC12073609; doi:10.3390/plants14091324)
Supplement: Supplementary file 1 [file plants-14-01324-s001.zip › plants-3549272-supplementary materials.pdf]

Supplementary Materials

RAW DATA

1) Blood pressure values (mm Hg)

PAS	NTR	SHR
	123,33	180
	121,66	164,66
	112	167,5
	112	145,33
	124,5	181,5
	117,33	187,66
	127	157
	117,66	175,5

PAD	NTR	SHR
	100,33	153
	105,66	131,33
	97,33	137
	95,5	127,33
	104,5	135,5
	101,66	145
	108	124,5
	100	133,33

2) Urinary analysis

Urine Volume (mL/ 100 g)

Days	NTR VEH						
1	0,32	0,41	0,17	0,34	0,46	0,44	0,77
2	0,97	1,43	0,7	0,86	0,9	0,81	1,54
3	1,62	2,12	1,43	1,62	1,67	1,37	2,69
4	2,78	3,48	2,06	2,44	2,48	2,41	4,23
5	4,27	4,61	2,59	3,47	3,53	3,33	5,38
6	5,08	5,63	3,04	4,26	4,15	3,89	6,35
7	5,73	6,66	3,67	4,78	4,95	4,26	7,04

Days	SHR VEH						
1	0,5	0,32	0	0,21	0	0,44	0,42
2	1,32	1,08	0,57	1,07	1,11	1,51	1,17

3	2,36	1,68	1,49	1,69	1,68	2,84	2
4	3,35	2,44	2,11	2,81	2,37	3,96	2,83
5	3,97	3,24	2,68	3,22	2,94	4,62	3,25
6	4,59	3,84	3,07	3,39	3,32	5,29	3,88
7	5	4,16	3,45	3,8	3,4	5,73	4,08

Days	HCTZ					
1	1,46	2,12	2,05	1,9	2,59	2,02
2	3,77	3,56	3,57	3,42	3,77	3,02
3	5,44	5,25	5,16	4,75	4,95	4,23
4	6,61	7,16	6,22	6,27	6,18	5,44
5	8,28	9,83	7,28	7,98	7,59	7,3
6	9,75	11,69	8,69	9,54	8,77	8,71
7	10,59	13,26	10,46	11,06	9,48	10,32

Days	HSP							
1	0,32	0,32	0,16	0,39	1,31	0	0,8	0,58
2	2,74	2,74	1,9	2,4	3,37	1,26	4,3	4,71
3	5,71	5,71	3,45	3,62	4,94	3,64	6,1	6,07
4	7,66	7,66	5,19	5,79	7,38	5,26	8,67	7,43
5	9,05	9,05	5,97	6,77	8,73	5,97	9,96	8,02
6	10,12	10,12	7,09	8,31	10,22	6,56	11,53	8,99
7	10,75	10,75	8,64	9,49	10,86	7,15	12,13	9,61

**Ca^{2+}**	VEH	VEH	HCTZ	HSP
	10,37409	11,06644	5,3378	5,85148
	11,41262	11,02178	4,623116	5,036293
	11,49079	7,816862	4,410944	4,768286
	9,704076	8,721385	4,477945	5,114461
	5,628141	8,096036	5,002792	5,974316
	9,112227	11,54662	4,835288	3,774428
	9,72641	10,4746		3,87493
				5,516471

**Na^{+}**	VEH	VEH	HCTZ	HSP
	68,73786	45	158,7866	96,78571
	106,4846	41,6	132,6271	86,4341
	51,3986	34,48276	94,13428	85,3937
	47,76632	45,61983	110,6464	119,4757
	59,44273	33,96947	94,81132	85,8498
	42,59259	57,33333	92,90323	109,1566
	84,46154	36,75		96,10895

			66,4898

Cl^-	VEH	VEH	HCTZ	HSP
	10,03236	13,20388	10,71197	6,957929
	8,899676	12,52427	10,64725	7,50809
	11,26214	13,3657	10,55016	6,601942
	15,17799	12,91262	11,42395	8,446602
	12,3301	13,07443	13,30097	7,89644
	10,74434	12,03883	10,87379	5,501618
	11,8123	13,52751		6,504854
				7,281553

K^+	VEH	VEH	HCTZ	HSP
	17,18447	32,5	27,52301	18,28175
	11,97952	24,96	18,5678	17,28682
	13,95105	20,68966	21,96466	15,1811
	20,53952	16,72727	22,12928	16,29214
	22,78638	21,74046	24,65094	8,58498
	11,5	24,65333	27,87097	21,83133
	21,11539	27,76667		13,45525
				8,126531

**Urea**	VEH	VEH	HCTZ	HSP
	8,37764	11,83765	9,7366	10,83752
	9,37762	14,8726	15,92887	9,36653
	5,37762	8,387613	11,93776	9,554173
	6,26637	8,45266	12,83776	12,63559
	8,7764	10,654	14,8876	8,665248
	4,82776	9,2665		11,82643
	6,2887	9,44537		9,73651
				8,26651

**Creatinine**	VEH	VEH	HCTZ	HSP
	3,98764	3,987465	2,384766	3,109938
	4,99876	4,098874	2,94876	2,009387
	3,64543	4,245536	3,00098	2,009376
	2,77654	5,24536	2,938873	1,938873
	4,009287	2,998465	2,11234	2,039987
	3,009187	4,982746		2,435627
	2,7455	3,99829		3,567489

				2,47765

**Uric acid**	VEH	VEH	HCTZ	HSP
	3,98764	3,987465	2,384766	3,109938
	4,99876	4,098874	2,94876	2,009387
	3,64543	4,245536	3,00098	2,009376
	2,77654	5,24536	2,938873	1,938873
	4,009287	2,998465	2,11234	2,039987
	3,009187	4,982746		2,435627
	2,7455	3,99829		3,567489
				2,47765

3) Blood analysis

**Ca^{2+}**	VEH	VEH	HCTZ	HSP
	1,44	1,33	1,38	1,34
	1,39	1,37	1,35	1,36
	1,29	1,38	1,37	1,36
	1,49	1,36	1,35	1,33
	1,51	1,43	1,36	1,34

**Na^{+}**	VEH	VEH	HCTZ	HSP
	140,3	139,2	140	138,5
	139,3	140,1	137,3	138,4
	133,1	138,6	138,7	138,5
	137,5	138,3	137	137,7
	137,4	141,3	141	138

**Cl^{-}**	VEH	VEH	HCTZ	HSP
	97,1	98,3	100,4	99,8
	97,6	98,6	98,6	97,2
	98,6	99,2	98,3	98,3
	96,5	97,8	97,4	98,8
	95,9	98,7	98,6	98,5

**K^{+}**	VEH	VEH	HCTZ	HSP
	4,77	5,17	5,51	5,94
	5,68	5,13	5,56	5,19
	6,75	5,99	5,33	5,44
	6,48	5,96	5,41	5,49
	5,86	5,69	5,45	5,5

**Urea**	VEH	VEH	HCTZ	HSP

	60,39	53,27	52,87	56,71
	59,4	50,01	54,26	53,63
	64,15	53,54	54,78	56,27
	56,39	50,32	55,83	51,18
	57,86	54,6	54	54,6

**Creatinine**	VEH	VEH	HCTZ	HSP
	0,29	0,23	0,22	0,24
	0,18	0,28	0,23	0,22
	0,37	0,17	0,23	0,2
	0,23	0,24	0,28	0,2
	0,22	0,23	0,24	0,24

**Uric acid**	VEH	VEH	HCTZ	HSP
	0,96	0,96	0,82	1,22
	1,42	0,97	1,23	0,9
	1,06	1,23	0,88	1,02
	1,39	1,28	0,93	1,36
	1,38	0,74	1,33	1,3

4) Crystals

Dihydrated CaOx

NTR VEH	SHR VEH	HCTZ	HSP
112	88	104	40
112	104	64	32
112	128	80	72
152	112	72	56
137	124	81	37
129	107		60
100	93		45
			58

Monohydated CaOx

NTR VEH	SHR VEH	HCTZ	HSP
24	16	24	0
24	8	16	0
32	8	32	0
48	40	0	0
26	14	20	0
30	19		0
40	21		0
			0

5) Histological Analysis

Corpuscle Area

NTR VEH	SHR VEH	HCTZ	HSP
3283115	2288275	2404346	3196378
2592304	2540697	1996922	2203350
1799909	1762245	3012042	3151471
1647080	4281394	3070530	2384427
1750475	1976641	2870258	2515527
2712540	2534359	2653508	1734359
2542327	1875238	2711272	3549842
1479765	1444273	2735899	3013852
1688004	1725668	2076596	2628339
1770575	2172929	2265278	3407153
3263015	1865822	3065641	2273608
2846899	1256134	1409325	3814215
2636125	1367497	2867542	3181892
2774830	3478859	1459122	2283205
2700951	3195473	2026981	2708918
2540516	2242282	3801177	2819737
2551562	1756813	2172205	2957356
2470982	2182707	3286193	3755545
2258397	2763603	3965414	2553191
3180444	2311634	3196378	1210321
2745315	1110729	2692259	2223631
3255048	3199819	2217293	2345677
2369217	3050792	3505115	2632141
2458307	2584337	3745043	3776732
1166863	2149208	6007424	1387234
2924762	2444907	3268628	2041105
2629787	2678135	2216931	853961
2704391	3295247	2749479	1648348
2945043	2627795	2735899	1548212
3234043	1625532	2241738	2019737
3081213	3358262	3822182	2309823
1479765	2908828	1405885	2119330
2135989	3057311	2607877	2534359
2784971	2574921	2553191	1391218
2496333	2304572	2714531	2146854
1761883	1982979	3091172	2618379
3070711	2573834	3524853	2366320
2352558	2532549	3485740	2115708
2479312	1774196	3660661	1614848
2129470	2196107	2946311	3033771
2627071	2470982	3943504	2218560
2972386	2260027	2609688	1560344
2115708	1955274	2147759	2158986
2417021	2428610	1418742	1785967
2594477	2747306	2119149	2347488
2129470	1913445	2043821	1441557
2784971	2904482	2534359	2708556
1812223	2241738	2237755	2419556
2748031	3589860	2031326	1974830
1637664	2488004	1828882	2480036

2458307	1200181	2040199	2311634
2217112	1554731	2000181	1849887
2444907	1816025	1423268	2383341
3028339	1046627	2578723	694613
3285287	3591852	2354912	1364418
2848710	2262019	1650702	2208963
2512630	1716614	2101947	2165686
2074604	2757447	1791218	1721503
1243640	1730376	2846899	2396741
2573834	2480036	2100498	2401449
2348212	2635401	1916161	1779267
2368674		1873246	1269353
2603893		2149932	2403078
2403259		1895880	1915075
2539430		2085287	2255138
2097239		1076505	1513083
2115527		1438479	1965052
2871163		2580715	2348574
2786419		2107741	4231417
2362698		2902671	2821910
1906383		2627071	2365052
3366410		1939701	3686917
3117429		3565414	2950113
2333364		2708918	2586691
2397284		2924762	2709461
2908828		2194658	3206519
2661114		3193481	2605704
2874242		3788864	2401086
2294613		4313083	2851426
2464101		2031689	2295518
1485559		2508465	3046084
2302580		2388049	1493526
1611589		2740244	2885650
2711272		2716523	1605432
1123404		2925487	2275962
		3411498	2214577
		2574196	2384427
		2856496	2058850
			2209144
			1839022

Bowman capsule

NTR VEH	SHR VEH	HCTZ	HSP
845813	257673	531100	418832
469352	304753	459213	337528
504119	151018	534360	244092
561521	210412	432956	258940
600271	242282	501041	469533
372839	277591	334631	490719
310367	331734	334088	621639
304029	214395	438751	479855
641377	213128	569308	387687
475509	317610	424807	648076

895971	622544	504844	395292
687913	201177	227252	549027
391670	272522	235401	438931
515346	223811	204618	470801
878588	309823	258217	266546
729380	300951	621096	357265
610231	119149	378814	509189
852512	165142	502128	641919
525124	366138	908827	538705
411770	214395	507378	311815
700589	157538	348755	391852
715618	279403	633952	265460
798914	475871	680126	512811
480398	405251	265097	513717
391127	474604	312539	418108
519149	465369	431145	162789
356541	503939	448167	93617
624174	494704	738071	155546
760525	425894	588140	281394
838027	231055	464282	166953
733725	484020	450702	265097
287913	161522	299864	220914
493255	427343	385514	365233
491807	352739	369397	326121
518424	566772	643549	321594
508103	428611	719782	297872
563151	248076	697148	324672
601902	345315	615482	250249
494704	402354	605885	320869
256224	316523	466999	539611
577999	392213	706383	191579
655319	648438	264011	195926
664191	258579	200634	252603
422997	618198	304754	215664
593934	411045	442734	250249
697510	283567	169308	163150
869172	718697	705477	351652
738796	244816	261114	235219
852513	520055	233589	284291
589588	376641	392214	398732
578723	257492	411951	246989
593210	216750	416116	192667
576912	245722	126754	334269
327388	111905	571118	273065
435853	646085	387687	481303
484382	446899	349118	533635
396922	543051	337167	285378
505568	483115	466275	367768
460299	362879	350566	501947
518968	482752	415754	598461
499231	402717	255681	230874
599910		247714	272884
517881		348936	487279
487460		362516	251879

580897		410683	400181
312540		285559	378090
394024		471163	336804
705477		301313	536895
670711		378814	858850
297329		474061	428791
438207		601539	400000
849253		524581	445450
834224		503033	473698
602083		553916	264554
562608		944681	507560
581802		436758	504482
621458		555907	183613
380444		880036	272702
333907		807062	515165
276867		458126	295337
441104		417021	685559
366500		276686	367043
327931		424626	844545
605523		445450	416478
289724		119511	343323
		753282	241739
		294794	572204
		392395	382435
			288818
			191942
